# The use of two-sample methods for Mendelian randomization analyses on single large datasets

**DOI:** 10.1093/ije/dyab084

**Published:** 2021-04-26

**Authors:** Cosetta Minelli, Fabiola Del Greco M., Diana A van der Plaat, Jack Bowden, Nuala A Sheehan, John Thompson

**Affiliations:** 1 National Heart and Lung Institute, Imperial College London, London, UK; 2 Institute for Biomedicine, Eurac Research, Bolzano, Italy; 3 Medical School, University of Exeter, Exeter, UK; 4 Department of Health Sciences, University of Leicester, Leicester, UK

**Keywords:** One-sample Mendelian randomization, two-sample Mendelian randomization, two-stage least square estimator, inverse-variance weighted estimator, weighted median estimator, weighted mode estimator, MR-Egger regression, UK Biobank

## Abstract

**Background:**

With genome-wide association data for many exposures and outcomes now available from large biobanks, one-sample Mendelian randomization (MR) is increasingly used to investigate causal relationships. Many robust MR methods are available to address pleiotropy, but these assume independence between the gene-exposure and gene-outcome association estimates. Unlike in two-sample MR, in one-sample MR the two estimates are obtained from the same individuals, and the assumption of independence does not hold in the presence of confounding.

**Methods:**

With simulations mimicking a typical study in UK Biobank, we assessed the performance, in terms of bias and precision of the MR estimate, of the fixed-effect and (multiplicative) random-effects meta-analysis method, weighted median estimator, weighted mode estimator and MR-Egger regression, used in both one-sample and two-sample data. We considered scenarios differing by the: presence/absence of a true causal effect; amount of confounding; and presence and type of pleiotropy (none, balanced or directional).

**Results:**

Even in the presence of substantial correlation due to confounding, all two-sample methods used in one-sample MR performed similarly to when used in two-sample MR, except for MR-Egger which resulted in bias reflecting direction and magnitude of the confounding. Such bias was much reduced in the presence of very high variability in instrument strength across variants ($I_{\text{GX}}^{2}$ of 97%).

**Conclusions:**

Two-sample MR methods can be safely used for one-sample MR performed within large biobanks, expect for MR-Egger. MR-Egger is not recommended for one-sample MR unless the correlation between the gene-exposure and gene-outcome estimates due to confounding can be kept low, or the variability in instrument strength is very high.

## Introduction

Mendelian randomization (MR) is an instrumental variable approach to investigate the causal effect of an exposure on an outcome by using genetic variants, typically single nucleotide polymorphisms (SNPs), as instruments for the exposure^1^; the causal effect is indirectly estimated from the gene-exposure and gene-outcome associations. The validity of MR relies on instrumental variable assumptions,^2^ the most problematic being the absence of pleiotropy whereby the genetic instruments modify the outcome only through the exposure of interest and not through any other independent pathway.

**Key Messages**

When full genetic, exposure and continuous outcome data are all available within the same study (‘one-sample MR’), the causal effect can be estimated using the two-stage least-square (2SLS) method; for a binary outcome, the analogue is a two-stage estimator with a logistic or log-linear model for the second-stage regression.^3^ When only summary statistics (β coefficients and standard errors) for gene-exposure and gene-outcome associations are available from separate studies (‘two-sample MR’), the causal effect is often estimated by first deriving SNP-specific causal effect estimates as the gene-outcome estimate divided by the gene-exposure estimate (Wald estimator), and then pooling them using inverse-variance weighted fixed-effect meta-analysis (IVW FE).^4^^,^^5^ Two-sample MR has been widely used to exploit summary data from large genetic consortia,^5^ and this has addressed the issue of low statistical power typical of MR.^6^ However, the two-sample approach has the important limitation of having to assume that the samples are homogeneous, so that the gene-exposure associations are identical across the samples. This may be violated in practice.^7^ Recently, large population-based biobanks have made available individual-level genome-wide data and data on a variety of exposures and outcomes, thus allowing well-powered one-sample MR studies. An important example is the UK Biobank (UKB), where individual-level data are publicly available for about 500 000 individuals aged 40–69.^8^

- Current availability of phenotypic and genetic data from large biobanks, such as UK Biobank, has led to increasing use of one-sample Mendelian randomization (MR) to investigate causal relationships in epidemiological research.
- Robust MR methods have been developed to address pleiotropy, but they assume independence between the gene-exposure and gene-outcome association estimates; this holds in two-sample MR but not in one-sample MR.
- We illustrate the practical implications, in terms of bias and precision of the MR causal effect estimate, of using robust two-sample methods in one-sample MR studies performed within large biobanks.
- Two-sample MR methods can be safely used for one-sample MR performed within large biobanks, expect for MR-Egger regression.
- MR-Egger is not recommended for one-sample MR unless the correlation between the gene-exposure and gene-outcome estimates due to confounding can be kept low, or the variability in instrument strength is very high.

Whereas both the 2SLS method for one-sample MR and the IVW FE method for two-sample MR assume no pleiotropy, several alternative methods that are robust to pleiotropy have been developed for two-sample MR.^9^ It is therefore tempting to use robust two-sample MR methods in a one-sample MR. The problem is that these methods assume independence between the gene-exposure and gene-outcome estimates, as would be the case when they are obtained from separate non-overlapping samples.^10^ This assumption does not hold in one-sample MR, where the two estimates are obtained from the same individuals and are therefore correlated.

Using simulations, we investigated the practical implications of applying methods that assume independence between the gene-exposure and gene-outcome estimates (referred to as ‘two-sample MR methods’) to a one-sample MR in the specific context of a large biobank, focusing on a continuous outcome. We considered the case where the choice of SNPs for the one-sample MR is based on evidence from previous studies, thus avoiding any issue with the winner’s curse.^11^ To inform our simulations, we reviewed 10 published studies that used two-sample MR methods in one-sample MR within UKB. In the simulations we assessed the performance, in terms of bias and precision of the causal effect estimate, of five different two-sample MR methods (fixed-effect and multiplicative random-effects meta-analysis, weighted median estimator, weighted mode estimator and MR-Egger regression), in both one-sample and two-sample data. We considered scenarios in which there was a true causal effect or not, in which the amount of confounding varied (implicit in the varying amount of correlation between exposure and outcome errors) and in which pleiotropy varied (none, balanced or directional).

## Methods

### Examples of published UKB studies

To inform our simulations, we searched PubMed on 25 April 2019 to identify examples of one-sample MR studies performed within UKB (‘UK Biobank[Title/Abstract] AND (Mendelian randomization[Title/Abstract] OR Mendelian randomisation[Title/Abstract]’). We reviewed the 10 most recent studies identified that had used two-sample methods with multiple instruments. From these we extracted information on sample size, number of instruments, variance of the exposure of interest explained by the instruments, F statistic and MR methods used in main and in secondary analyses. For studies using MR-Egger, we also recorded the variability in instrument strength, expressed as heterogeneity in gene-exposure estimates across SNPs ($I_{\text{GX}}^{2}$), since we have previously shown that MR-Egger works well only when this is large, with recommended $I_{\text{GX}}^{2}$ over 90%.^12^ Although 110 eligible MR investigations were reported within the 10 papers, for each paper we only considered MR analyses performed on different exposures (different sets of instruments) or population subgroups (different sample sizes). This resulted in 27 MR investigations, whose characteristics are summarized in Supplementary Table S1 (available as Supplementary data at *IJE* online). The sample size varied from 180 957 to 376 435 (median: 318 664), the number of instruments from 2 to 520 (median: 68), and the variance explained, reported in five studies, from 0.2% to 7.3% (median: 1.8%). Only three studies reported the F statistic; of these, only one study (reporting on four MR investigations) provided F values for all SNPs, which varied from 10.1 to 382.5.

The most commonly used two-sample methods were: MR-Egger regression (*N* = 10 studies); weighted median estimator (*N* = 9); IVW (*N *= 8), mostly as the main analysis, with only three studies specifying whether a fixed-effect or a random-effects model was used; and weighted mode estimator (*N* = 3). All studies used MR-Egger but none reported an $I_{\text{GX}}^{2}$ value, although one mentioned the limited variability in instrument strengths as an explanation for the limited power of the MR-Egger analysis.^13^ A one-sample MR method was also used in three studies: 2SLS (*N* = 2) and a maximum likelihood method^3^ (*N* = 1).

### Methods for the simulations

Mirroring a typical analysis using UKB, the simulations created data on 300 000 individuals and 100 independent SNPs with allele frequencies between 1% and 99%. Both the exposure, X, and the outcome, Y, were continuous with normally distributed errors, and all relationships between X, Y and the genotype, G, were linear with no interactions. SNP coefficients for the G-X association were simulated using an exponential distribution, resulting in many SNPs with small effects and a few with large effects (Supplementary Methods, available as Supplementary data at IJE online). The individual SNPs had an average F statistic of 67.9; only 5% of the SNPs had an F statistic below 10 and would generally be considered as weak.^14^ On average, the 100 SNPs explained 2.3% of the variance in X. The average $I_{\text{GX}}^{2}$ was 91%.

We simulated data with no causal effect of X on the outcome, Y, and data where the causal effect was 1.0. A causal effect of 1.0 was strong enough for the 100 SNPs to explain 0.2% of the variance of the outcome in the absence of pleiotropy. For each scenario, different degrees of confounding between X and Y were simulated by generating a correlation between the error components in X and Y of −0.4, −0.2, 0, 0.2 and 0.4 (a correlation of 0 represents no confounding).

As well as the situation of no pleiotropy, we simulated data in which 20% of the SNPs were pleiotropic (details in Supplementary Methods). The pleiotropic effects were generated from the same distribution as the G-X effects but independently, so that the InSIDE (Instrument Strength Independent of Direct Effect) assumption needed for the IVW and MR-Egger methods holds.^15^ For balanced pleiotropy, the pleiotropic effects were given a random sign so that the average was zero, whereas for directional pleiotropy, the effects were all positive.

For comparison, genuine two-sample data were also simulated by creating two datasets under identical conditions and taking the G-X estimates from one and the G-Y estimates from the other. In order to generalize our findings beyond UKB, we also performed a secondary analysis where we considered a smaller sample size of 100 000.

All simulations were run 1000 times. Further details of the simulation parameters are reported in Supplementary Methods, and the R code used for the simulations is available at [https://github.com/thompson575/2in1].

### MR methods compared

The two-sample methods investigated were IVW FE^5^ and four methods robust to pleiotropy, which are based on different assumptions about its nature: multiplicative random-effects meta-analysis (IVW RE), that has been recommended over the additive RE model (see Supplementary Methods)^16^; weighted median estimator^17^; weighted mode estimator^18^; and MR-Egger regression.^15^ In the one-sample MR we also calculated the 2SLS estimator^3^ which represents the gold standard in the absence of pleiotropy. Further details are reported in Supplementary Methods.

All two-sample methods were implemented using the MendelianRandomization R package version 0.4.3 [https://CRAN.R-project.org/package=MendelianRandomization] and for 2SLS we used the AER R package version 1.2–9 [https://CRAN.R-project.org/package=AER].

## Results

Overall, the results of our simulations show that, except for MR-Egger, two-sample methods perform similarly when applied to a one-sample MR with a large sample size (300 000 in our simulations) or a two-sample MR of the same size. In the presence of confounding between X and Y, MR-Egger used in one-sample MR gives biased results that reflect the magnitude and direction of the confounding; in particular, the bias is in the direction of the observational association, which can be viewed as the sum of the causal effect of X on Y, and the confounder effect (see causal diagram in Supplementary Methods).

**Figure 1:**
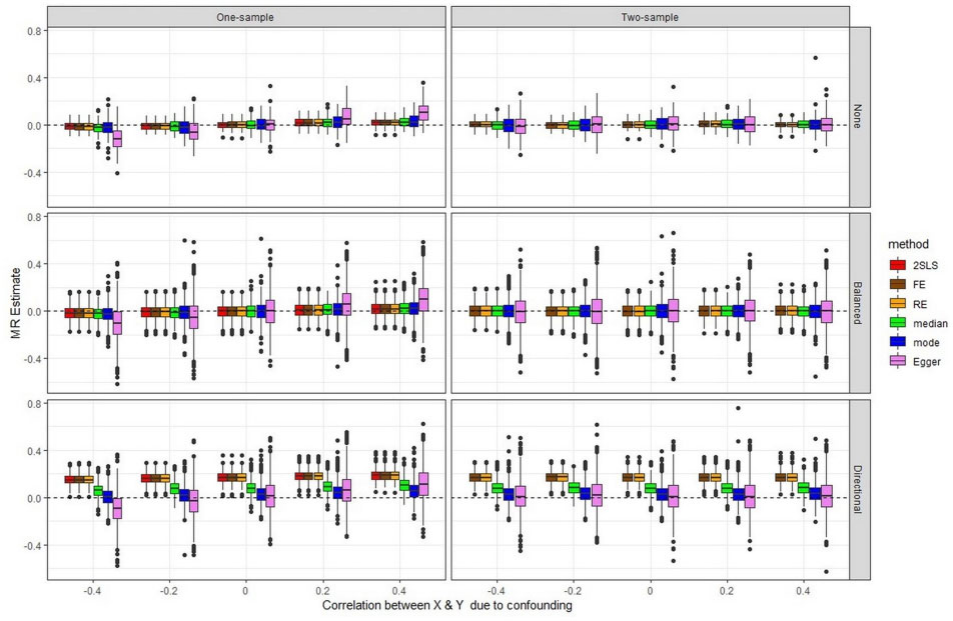
Simulations with no causal effect: box plots summarizing the results of all methods across the 1000 simulations, in scenarios with no pleiotropy, balanced pleiotropy and directional pleiotropy, and for both one-sample and two-sample MR. Outliers have been removed (see text)

**Figure 2:**
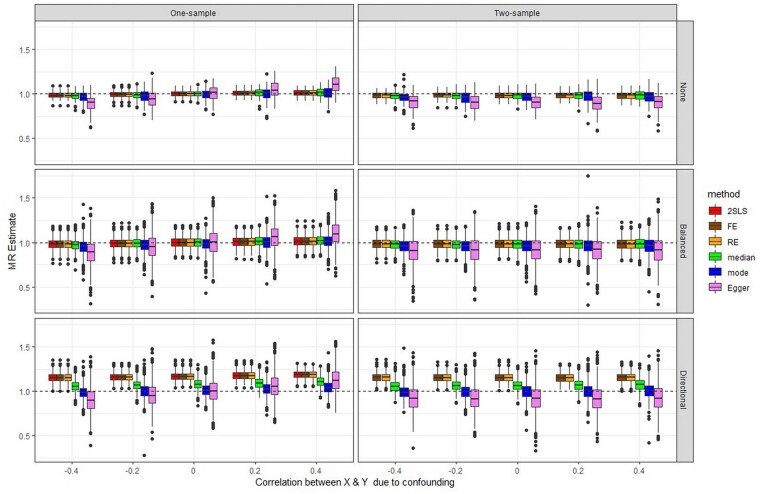
Simulations with a causal effect of 1: box plots summarizing the results of all methods across the 1000 simulations, in scenarios with no pleiotropy, balanced pleiotropy and directional pleiotropy, and for both one-sample and two-sample MR. Outliers have been removed (see text)

Figures 1 and 2 show boxplots of the 1000 MR estimates generated when there is no true causal effect and with a causal effect of 1, respectively. The point estimates for the causal effect from IVW FE, IVW (multiplicative) RE and 2SLS are asymptotically identical,^19^ as reflected in Figures 1 and 2. Numerical results corresponding to Figures 1 and 2, expressed as mean, standard error, coverage and root mean square error (RMSE) of the causal effect, are reported in Supplementary Tables S2-44 (available as Supplementary data at *IJE* online) for each pleiotropy scenario. To improve readability of the plots, outliers (values more than 0.8 above/below the true value) were removed from the graphs, but not from the results in Supplementary Tables S2-4. Supplementary Table S5 (available as Supplementary data at *IJE* online) includes the ordinary least squares (OLS) estimate for regression of Y on X in one-sample data, which reflects the amount of confounding present in the simulations.

### Scenarios with no pleiotropy

**Figure 3:**
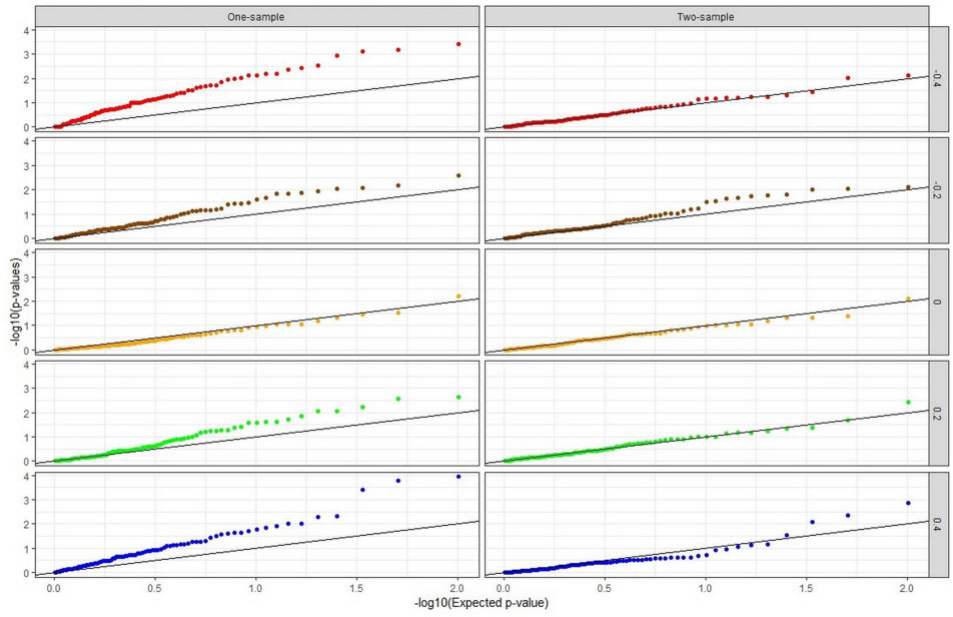
Simulations with no causal effect and no pleiotropy: Q-Q plot of MR-Egger null *P*-values [plot of -log_10_(*P*-value) against its expectation across simulations] for different levels of correlation between X and Y due to confounding, in one-sample and two-sample MR

The top panels of Figures 1 and 2 present the results for the scenarios with no pleiotropy. In the absence of a causal effect (Figure 1), all two-sample methods applied to one-sample MR (left) give the same results on average as when applied to a genuine two-sample MR (right) in the absence of confounding, since the G-X and G-Y estimates become independent even in a one-sample MR. In the presence of confounding, all two-sample methods other than MR-Egger show only minimal bias in the direction of the confounding when applied to one-sample MR; the fact that 2SLS shows the same minimal bias suggests some weak instrument effects affecting all methods. For MR-Egger, however, the bias in the direction of the confounding is substantial. The *P*-values of MR-Egger analysis are shown in the Q-Q plots of Figure 3 (corresponding Q-Q plots for scenarios with balanced and directional pleiotropy in Supplementary Figure S1, available as Supplementary data at *IJE* online). Figure 3 shows increasing departure of the observed from the expected *P*-values under the null with increasing confounding when MR-Egger is applied to the one-sample MR (left), but not in a two-sample MR (right). Since the points are above expectation, in the presence of confounding towards an observational estimate more extreme than the true causal effect, MR-Egger in one-sample MR will mislead by indicating a significant causal effect more often than it should.

Findings for the scenarios with a true causal effect (Figure 2) tend to follow the same pattern as for those with no effect. In the presence of confounding, all two-sample methods used in one-sample MR show a very small bias in the direction of the confounding, except for MR-Egger where this bias is substantial. Interestingly, all methods applied to a genuine two-sample MR show a bias towards the null, but this bias is of very small magnitude except for MR-Egger where it can be very pronounced. Further investigation (data not shown) indicated that this is in part due to regression dilution bias, which for MR-Egger is accurately quantified by the $I_{\text{GX}}^{2}$ statistic that measures the sample variation in instrument strength. This is in line with our previous findings of this bias being much more marked for MR-Egger than for the IVW approach.^12^

To further investigate the behaviour of MR-Egger in the one-sample MR, in a secondary analysis we repeated the simulations after increasing the variability in instrument strength, from a value of $I_{\text{GX}}^{2}$ of 91% to 97%. The bias in the one-sample MR remained in both scenarios of absence and presence of a causal effect (Supplementary Figure S2a and b, respectively, available as Supplementary data at *IJE* online), but its magnitude was substantially reduced.

### Scenarios with pleiotropy

When there is balanced pleiotropy, as expected all methods produce more variable causal effect estimates in both one-sample and genuine two-sample MR, and once again MR-Egger gives the most variable estimates. In general, in both the absence and the presence of a causal effect (Figures 1 and 2), we observed the same trends as with no pleiotropy, except for the weighted median and weighted mode estimators that were here more similar to the 2SLS, IVW FE and IVW RE methods in terms of variability. 2SLS and IVW FE, which do not allow for pleiotropy, yielded estimated standard errors that were too small, resulting in lower coverage and exaggerated significance, whereas the other methods gave reasonable estimated standard errors and coverage (Supplementary Table S3).

Under directional pleiotropy, as expected the 2SLS, IVW FE and IVW RE methods perform badly in terms of bias across all scenarios (absence/presence of a causal effect; one-sample/two-sample MR; all levels of confounding; Figures 1 and 2). The weighted median estimator tends to be more biased than the weighted mode estimator and MR-Egger across all levels of confounding, and for both one-sample and two-sample MR. This behaviour of the weighted median estimator was unexpected for a genuine two-sample MR, particularly given that directional pleiotropy was simulated for only 20% of the SNPs. As a consequence of bias accompanied by a small standard error, the coverage for 2SLS, IVW FE and IVW RE is very low (1% to 17% instead of 95% across all one-sample MR scenarios; Supplementary Table S4); for the weighted median estimator, the coverage tends to be worse than that of the weighted mode estimator and MR-Egger. Although in general MR-Egger performs well under directional pleiotropy, the same pattern of bias in the direction of the confounding is observed when applied to the one-sample MR, whereas in a genuine two-sample MR it shows bias towards the null (Figure 2). It should be considered that in our simulations only 20% of the SNPs are pleiotropic, and this disadvantages MR-Egger which assumes that pleiotropy is spread across all SNPs.

### Results of the secondary analysis with smaller sample size

The results of the secondary analysis with a smaller sample size of 100 000 are reported in Supplementary Figure S3a and b (available as Supplementary data at IJE online) for when there is no true causal effect and with a causal effect of 1, respectively.

In the scenarios with no pleiotropy, MR-Egger used in one-sample MR shows the same trend with bias in the direction of the confounding as in the main analysis (*N* = 300 000; Figures 1 and 2), but the bias here is more pronounced. For all other two-sample methods used in one-sample MR, the bias in the direction of the confounding is more evident than in the main analysis but still small. All methods applied to a genuine two-sample MR show a bias towards the null in the presence of a true causal effect. Similarly to the main analysis (Figure 2), this bias towards the null is pronounced only for MR-Egger, but here it is more evident also for the other methods although it remains small (Supplementary Figure S3b).

In the scenarios with pleiotropy, the differences with the main analysis reflect those of the scenarios with no pleiotropy for two-sample methods used in one-sample MR. In a genuine two-sample context, MR-Egger shows bias towards the null even when used under directional pleiotropy (Supplementary Figure S3b), and the bias is much more pronounced than in the main analysis.

It is important to note that the smaller sample size in these secondary analyses implies not only less precision but also a higher proportion of weak instruments (a third of the SNPs compared with only 5% in the main analysis), and both these aspects could have influenced the performance of the methods.

## Discussion

With genome-wide association data for many exposures and outcomes becoming readily available from large biobanks such as UK Biobank, one-sample MR is being increasingly used to identify and estimate causal relationships. The main obstacle to the validity of MR is the presence of pleiotropy; several methods robust to pleiotropy are available, but these have been developed for two-sample MR. Our study evaluated the practical implications of using two-sample methods for one-sample MR, where the assumption of independence between the gene-exposure and gene-outcome estimates measured in the same individuals does not hold in the presence of confounding. We show that most two-sample methods, in particular the fixed-effect and (multiplicative) random-effects meta-analysis, the weighted median estimator and the weighted mode estimator, perform well when used in one-sample MR applied to a large sample size, even in the presence of substantial confounding. However, MR-Egger regression does not, with bias in the direction of the confounding increasing with the magnitude of the induced correlation. Our findings confirm previous suggestions of a bias of the MR-Egger estimate towards that of the confounded observational association in the one-sample setting, that has been attributed to the presence of weak instruments to which MR-Egger is particularly vulnerable.^20^^,^^21^ MR-Egger may suggest a causal effect when in fact there is none when used in one-sample MR in the presence of confounding, with a positive or negative spurious effect according to the direction of the confounding. Therefore, as opposed to what happens in a two-sample MR, MR-Egger is not necessarily conservative when used in the one-sample MR, since type 1 error is also affected in the presence of confounding. Under directional pleiotropy, although as expected MR-Egger performed better, we observed the same pattern of bias: the weighted mode estimator appeared to be a preferable option in one-sample MR in the scenarios considered in our simulations. It should be noted that our choice of simulating pleiotropy for only 20% of the SNPs disadvantages MR-Egger compared with a situation of directional pleiotropy where most of the SNPs are pleiotropic, which is when MR-Egger performs at its best. As for the behaviour of the weighted median estimator under directional pleiotropy, the reason for the biased results even when applied to a genuine two-sample MR is unclear, given that the pleiotropy affected only 20% of the SNPs; further research is needed to investigate this behaviour.

The bias for MR-Egger used in one-sample MR is much attenuated when the method is used at its best, that is with a maximum variability in instrument strength across variants that can be measured with the $I_{\text{GX}}^{2}$. However, we show that $I_{\text{GX}}^{2}$ needs to be much higher than the recommended 90%^12^ in order to reduce the bias in MR-Egger; in our simulations, substantial reduction was obtained by increasing it from 91% to 97%. Increasing the $I_{\text{GX}}^{2}$ threshold recommended for MR-Egger when used in one-sample MR could be an option, although in practice this may limit the application of the method. An easier option would be to reduce the confounding between exposure and outcome as much as possible, since this would reduce the correlation between the gene-exposure and gene-outcome estimates. In a given one-sample MR study, the observed correlation between the two estimates will be induced by both the true causal effect and the confounding; we therefore provide a simple way to disentangle the two and estimate the latter, which is what should be monitored (see derivation of ‘residual correlation’ under Parameters monitored, Supplementary Methods). In practice, the problem with this is that adjustment for potential confounders is not necessarily desirable; in fact, extensive adjustment is not recommended in MR as this may bias estimates if the variable adjusted for represents a mediator (on the causal pathway from the genetic variants to the outcome) or if the adjustment induces collider bias.^22^

Our findings show that, in a one-sample MR, the correlation between the gene-outcome and the gene-exposure estimates in the presence of confounding does not only affect the standard error of the MR-Egger estimate, as expected, but also biases the causal estimate in the direction of the confounding. However, the reason for this behaviour of MR-Egger in a one-sample setting is unclear and requires further investigation.

We focused on the use of two-sample methods in one-sample MR studies performed within large biobanks, such as UK Biobank, and the problems that we observed will be the more severe the smaller the sample size is. Our secondary analysis with sample size reduced to 100 000 shows a worse performance for all methods compared with the main analysis on 300 000 individuals. The bias in the direction of the confounding becomes even larger for MR-Egger; for all other methods this bias remains small but becomes more apparent, and we would expect this to become non-negligible with sample sizes smaller than 100 000. The impact of the size of the sample on the performance of the methods has to be interpreted in relation to the strength of the instruments used and thus the number of weak instruments, as increasing the magnitude of the effect of the SNPs on the exposure might compensate for a reduction in sample size, and vice versa.

This study has limitations that need to be addressed with further methodological work. When genetic variants are considered one at a time, as in all two-sample methods, other variants with a pleiotropic effect will induce further confounding between exposure and outcome. Although reflected in our simulations, this confounding cannot be disentangled from the other two sources of correlation (classical confounding and causal effect) in any given one-sample MR study; however, such confounding is very likely to be small. Moreover, our simulations for the pleiotropy scenarios assumed independence of the pleiotropic effects from the effects of the instruments on the exposure (InSIDE assumption); in practice, violations of this assumption would also contribute to create a correlation between the gene-exposure and gene-outcome estimates.

In conclusion, fixed-effect and (multiplicative) random-effects meta-analysis, weighted median estimator and weighted mode estimator are two-sample MR methods that can be safely used for one-sample MR studies performed within large biobanks, such as UK Biobank. On the contrary, the use of MR-Egger regression for one-sample MR is not recommended unless the correlation between the gene-exposure and gene-outcome estimates induced by confounding can be kept low, or the variability in instrument strength across variants is very high. Further work is required to correct for the bias in the MR estimate when MR-Egger is used in the one-sample setting.

## Supplementary data

Supplementary data are available at *IJE* online.

## Funding

None.

## Conflict of interest

None declared.

## Supplementary Material

dyab084_Supplementary_Data
